# A product-driven system with an evolutionary algorithm to increase flexibility in planning a job shop

**DOI:** 10.1371/journal.pone.0281807

**Published:** 2023-02-16

**Authors:** Patricio Sáez, Carlos Herrera, Camila Booth, Sana Belmokhtar-Berraf, Victor Parada

**Affiliations:** 1 Department of Industrial Engineering, Universidad de Concepción, Concepción, Chile; 2 Université Gustave Eiffel, ESIEE Paris, COSYS-GRETTIA, Marne-la-Vallée, France; 3 Department of Informatics Engineering, Universidad de Santiago de Chile, Santiago, Chile; 4 Instituto Sistemas Complejos de Ingeniería (ISCI), Santiago, Chile; TU Wien: Technische Universitat Wien, AUSTRIA

## Abstract

The scheduling of a job shop production system occurs using models to plan operations for a given period while minimizing the makespan. However, since the resulting mathematical models are computationally demanding, their implementation in the work environment is impractical, a difficulty that increases as the scale problem grows. An alternative approach is to address the problem in a decentralized manner, such that real-time product flow information feeds the control system to minimize the makespan dynamically. Under the decentralized approach, we use a holonic and multiagent systems to represent a product-driven job shop system that allows us to simulate real-world scenarios. However, the computational performance of such systems to control the process in real-time and for different problem scales is unclear. This paper presents a product-driven job shop system model that includes an evolutionary algorithm to minimize the makespan. A multiagent system simulates the model and produces comparative results for different problem scales with classical models. One hundred two job shop problem instances classified as small, medium, and large scale are evaluated. The results suggest that a product-driven system produces near-optimal solutions in short periods and improves its performance as the scale of the problem increases. Furthermore, the computational performance observed during the experimentation suggests that such a system can be embedded in a real-time control process.

## Introduction

Industry 4.0 requires manufacturing systems to be flexible, dynamic, and able to react immediately to disruptions. These are critical aspects in the design stage, for which it is necessary to resort to modeling that integrates the advantages of traditional modeling [1]. Such models are the basis of the manufacturing process control system [2], and should consider the integration of historical data with data from the system in real-time. Thus, dynamic decision-making should occur to reach a steady state of optimality. However, the control system must provide dynamic and near-optimal solutions in a reduced computational time.

The design of production systems has benefited from information and communication technologies, allowing the emergence of models with the capacity for self-learning, self-diagnosis, self-adaptation, and self-optimization [3]. Although there is a wide variety of production systems, job shop systems constitute a significant part, and their scheduling to optimize resources is a computational challenge [4]. The job shop scheduling problem (JSSP) emerges from that situation as a problem belonging to the NP-hard class [5]. Consequently, the computational requirement precludes its practical use in large-size situations. JSSP has been widely studied, visualizing a centralized and static approach. However, the integration of current technologies broadens the conception of production systems, giving rise to more sophisticated organizational ideas [1]. Thus, the JSSP can be approached in a decentralized and dynamic way, achieving that the flow of products during the operation contributes with real-time information that supports the decision-making of the production control system.

The development of intelligent systems makes it possible to meet the computational challenge offered by production systems dynamically. Such systems occur in different sizes, making it difficult to use a single method for production scheduling [6, 7]. Exact methods for JSSP cannot always be used in practice due to their computational cost; thus, it is necessary to resort to heuristic methods that produce a solution sacrificing optimality [8]. Product-driven systems (PDS) face such difficulties for JSSP. They are systems that consider the information coming from the product cycle to support decision-making in the control system. Then, the products are equivalent to agents actively participating in the control system [9, 10].

The advantage of using a PDS lies in the increased agility and reactivity of the production system. A PDS can react to disturbances related to itself or other parts of the manufacturing system [11]. Furthermore, the PDS considers product intelligent artificial entities to implement and coordinate the control process. Such products allow the reconfiguration of resources to provide agility in the face of production changes [12]. The PDS implementation occurs by applying concepts of a holonic manufacturing system (HMS) with a multiagent system (MAS). An HMS has a fundamental unit called a holon, which describes an entity in its physical and virtual forms. HMSs are not simple automated physical structures but entities capable of autonomous self-organization, mixing the physical and virtual worlds to avoid waste and inefficiencies [13]. In turn, a MAS constitutes a form of development based on the distribution, autonomy, and cooperation of virtual entities called agents [14]. Consequently, a PDS dynamically addresses the optimization of the JSSP [15].

Several studies demonstrate the benefits and difficulties of using a PDS when decision-making is decentralized. Mihoubi et al. [16], found good performance in minimizing production system execution times. In turn, Bouazza et al. [17], considered a PDS as a decision strategy for efficient scheduling rule changes using a hyperheuristic and found good performance in minimizing execution times. Shen and Norrie [18], used agents as negotiating entities, emphasizing flexibility by combining a MAS with a genetic algorithm. Likewise, Wang and Choi [19] presented a proposed holonic decomposition to minimize the makespan of a flexible JSSP. The proposed method uses autonomous and cooperative holons to construct solutions. In turn, the decentralized decision-making process generates difficulty due to the number of possible combinations of products and rules. The best combination in each event can be determined heuristically with a genetic algorithm but with an increased computational cost.

A PDS that integrates an HMS with a SAM, and contemplates the local optimization of decisions, gives rise to a large job shop production model. Its computational tractability must be considered for real life industrial applications. That is, the system must run in real-time with low computational times providing near-optimal solutions at every instant of the production process. Such efficiency considerations have received little attention in the literature; consequently, there is no clarity on its computational performance against problems of different sizes.

This manuscript proposes a PDS model that considers parameters associated with the HMS and MAS to increase flexibility in planning a Job Shop production system. The model produces a fast response with a near-optimal solution for problems of different sizes. In addition, the model considers intelligent products to support decision-making by considering a function based on evolutionary algorithms. The use of evolutionary algorithms brings more efficiency to the search process by modifying the representation of the modeled system. The model’s performance was tested with JSSP instances studied in the literature and compared with an integer programming model, a heuristic method, and dispatching rules. A comparison with this method allows us to analyze the robustness of the proposed model. Dispatch rules offer a fast alternative solution, although with lower accuracy. In turn, heuristics that are more elaborate methods obtain better solutions. Exact models guarantee the determination of the optimal solution but with a high computational cost.

The main contributions of this manuscript are as follows:

- It proposes a new model for the production planning of a job shop system. The model includes an evolutionary algorithm to search for better solutions applied through intelligent products.
- The proposed model uses the holonic-multiagent paradigm with intelligent products for decision-making in a highly distributed architecture. The product agents can apply evolutionary algorithms to optimize perturbed outcomes.
- Experimentation is performed on JSSP instances used in the literature.
- The model is adaptive in solving JSSP of different sizes and obtains near-optimal results in short execution times.

## Proposed model

This section describes the model proposed to solve the JSSP and the algorithm used to determine the optimal solution. This model is a product-driven system with an evolutionary algorithm (PDS-EA) that represents a productive job shop system in which machines and products interact through production jobs or operations. The process corresponds to an MAS that considers machines and products as agents of the system. In the holonic model, the machine agent and the product agent correspond to a virtual representation of their physical entities. The JSSP solution search occurs with an evolutionary algorithm operated by the product type agents. This process is generic and useful for representing several real industry situations.

**Fig 1:**
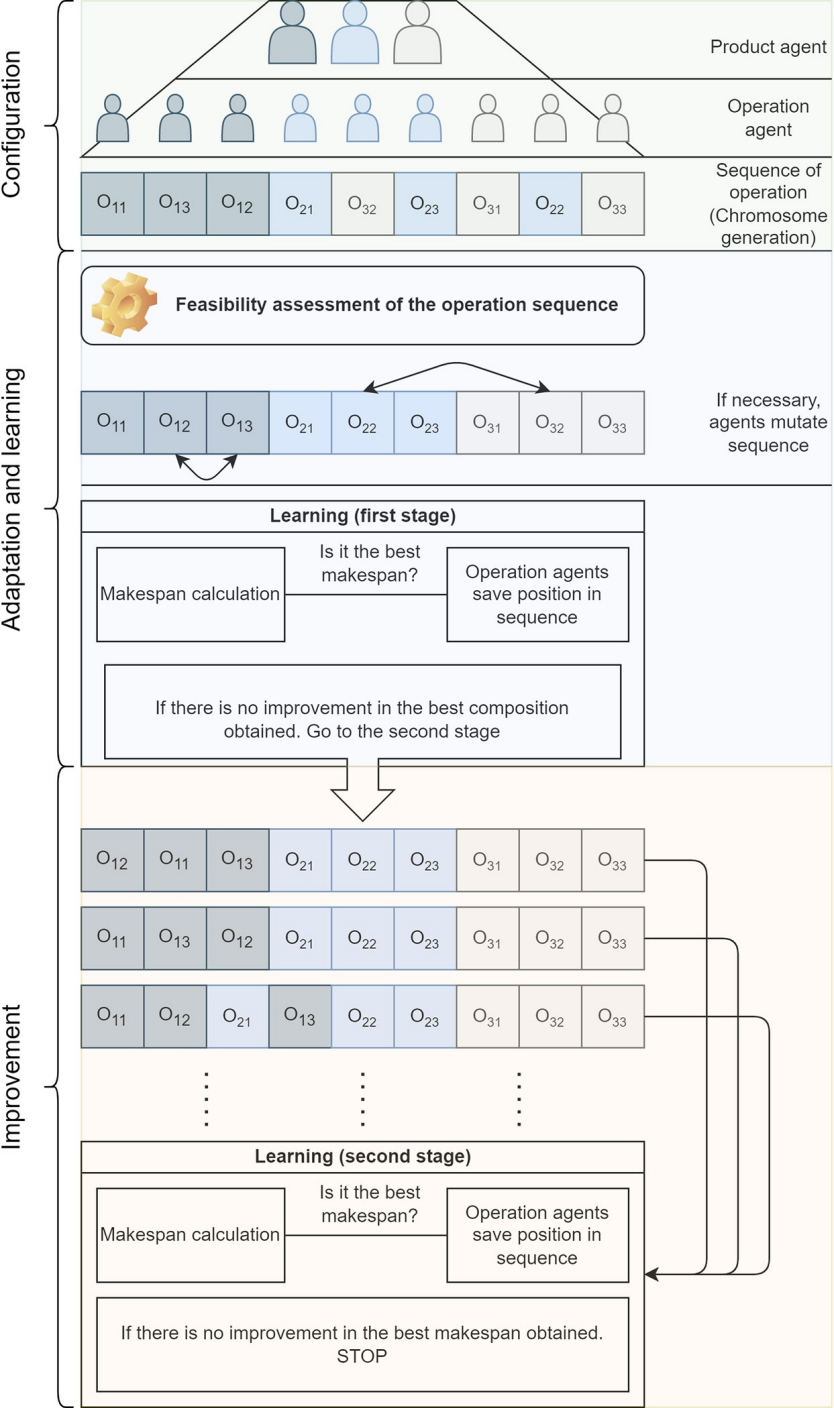
Execution procedure of the proposed approach.

Operating agents that possess the characteristics of an intelligent product, defined in the work of C. Wong et al. [20], represent the system’s mobile entities. That means they have a unique identification, can communicate with machines around them and other operation agents, and make operational decisions on a machine. In addition, machine agents are considered static entities that only provide information about the processing time of their tasks. Fig 1 depicts a scheme of the general model that follows an example selected from the literature [21]. The example considers the scheduling of three products on three machines, as shown in Table 1. Fig 1 show, in the first stage, the model configured through the product and operation agents, configuring the processing sequence of each of them. In the second stage, the operation agents generate feasible production sequences and calculate the associated makespan. In this stage, the operation agents learn the sequences that generate the best results. The best results from the second stage improve through an evolutionary process in the third stage.

**Table 1:** A problem instance with three products and three machines.

	M1	M2	M3
**J1**	*O*_11_	*O*_12_	*O*_13_
**J2**	*O*_21_	*O*_22_	*O*_23_
**J3**	*O*_31_	*O*_32_	*O*_33_

**Fig 2:**
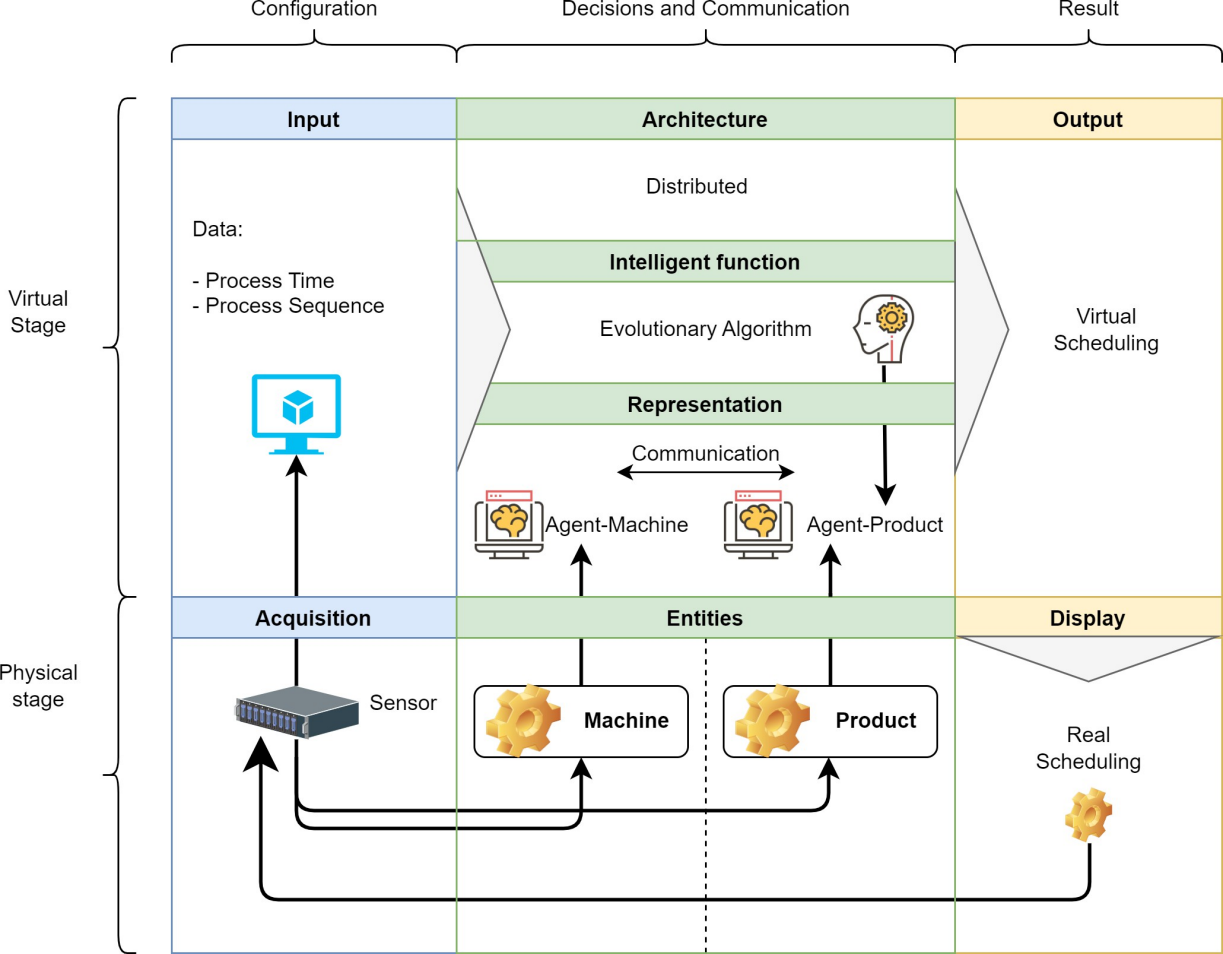
Schematic presentation of PDS/EA.

The PDS-EA model can be represented schematically by separating the physical and virtual stages. Both stages are represented horizontally in the diagram in Fig 2; the columns represent the elements of the model that, in the physical part, correspond to acquisition, entities, and visualization. In turn, the virtual part represents the information inputs, architecture, intelligence function, representation of the entities, agent’s response to the problem, interaction, and results.

The information flow of the PDS-EA model starts at the acquisition modules that represent physical sensors. Such modules capture information and transform it into data for each virtual entity. The entities represented virtually by agents follow a distribution given by the highly distributed architecture. In this architecture, all the entities associated with the products guide the decisions of their process. Such decisions are generated based on an intelligence function embedded in each agent to evaluate individual and collective performance. This evaluation occurs through three stages: data collection and reading, solution learning and improvement, and scheduling generation. In the data collection and reading stage, product and machine agents collect sequence data and operation times required to complete jobs. In the learning and improvement stage, the operation agents calculate the makespan of the problem through the intelligence function.

The PDS-EA model uses an intelligence function that is an evolutionary algorithm with an elitist selection strategy for makespan minimization. The advantage of using an elitist strategy instead of a probabilistic reproduction is that the best solution improves monotonically. The potential disadvantage is the convergence of the population to a local minimum. The balance between the two aspects occurs by regulating the mutation rate. Thus, a mutation evolved on a single chromosome is proposed instead of a gene-by-gene mutation. This process avoids the violation of the production sequence of each product in the JSSP.

**Fig 3:**
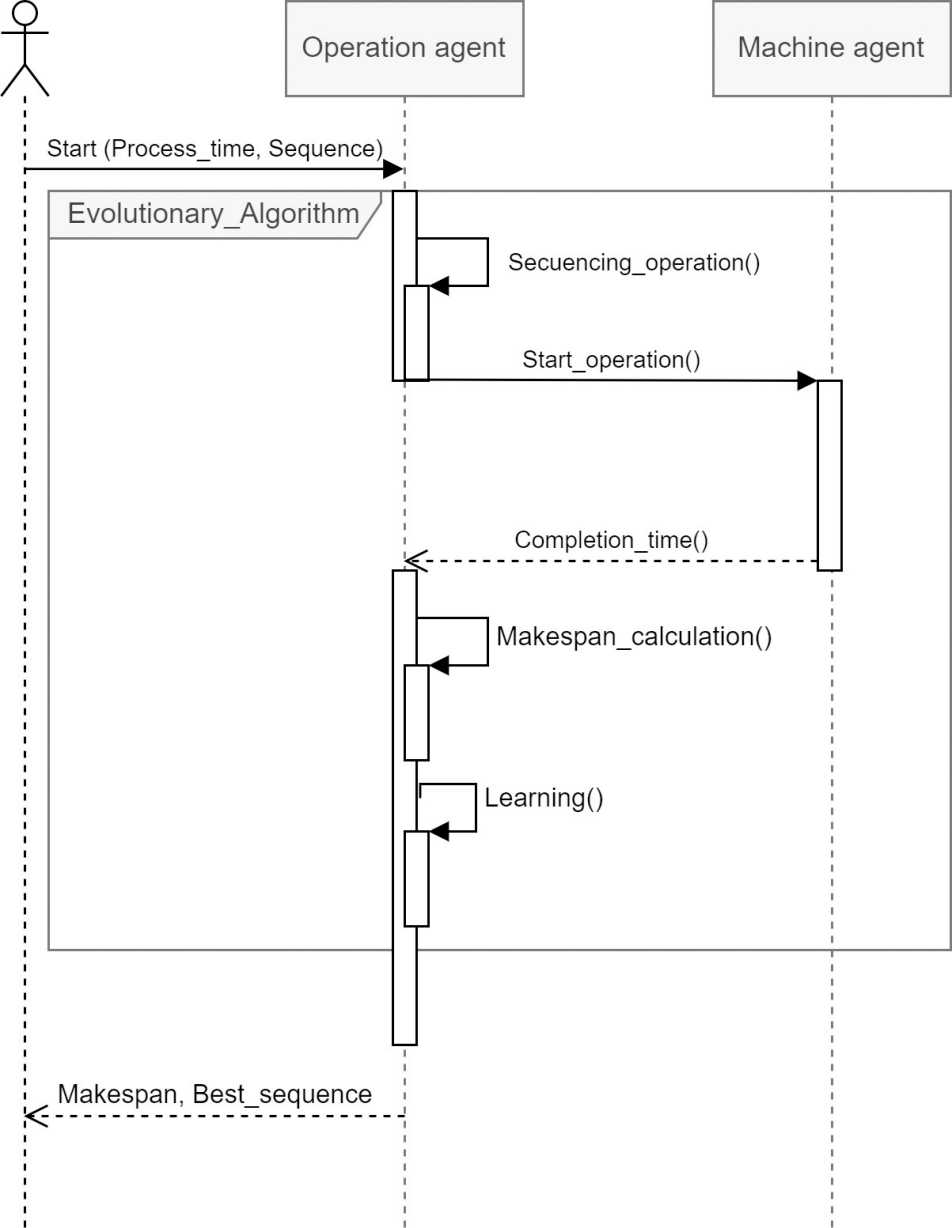
Diagram of the sequence for the action of the product agent.

**Fig 4:**
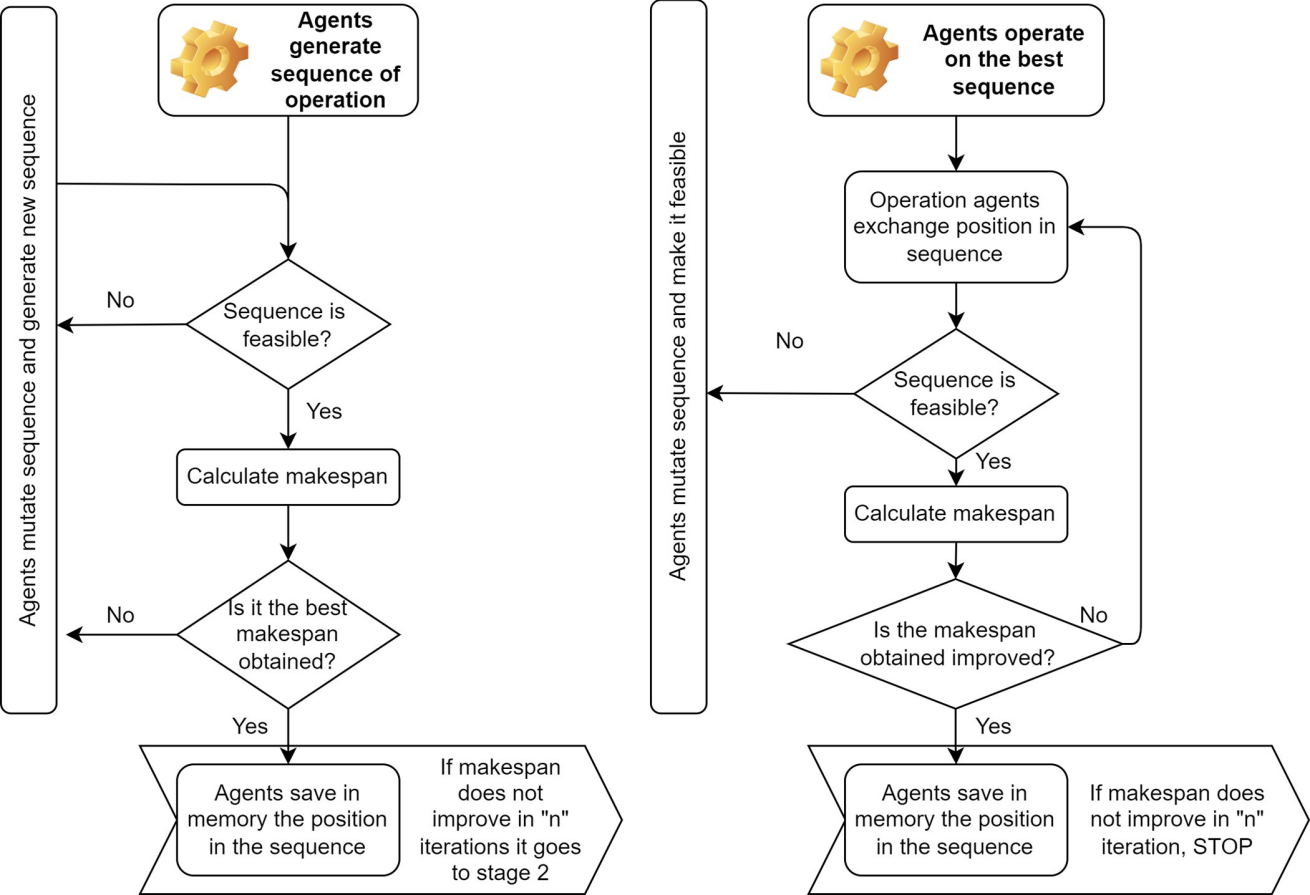
Flow diagram for the intelligent process in product decision-making.

The development of the actions of the PDS-EA model is represented through an UML-type sequence diagram. Fig 3 depicts the order of actions performed by the operation agents. The first decision occurs with the sequencing of operations generated by all product agents. Then, the operation agents evaluate the sequence’s feasibility, verifying the assignment of jobs to the machines according to the JSSP. If the generated sequence is not feasible, mutations are performed to make the sequence feasible. The makespan is then evaluated. If this improves with subsequent iterations, the product agents store the position in the generated sequence in memory and use it to minimize the makespan. Suppose there is no change in the best makespan after generations. In that case, a second stage begins in which the operation agents evaluate new sequences through an evolutionary swapping mechanism between the operation agents’ positions. If the operation agent generates an unfeasible sequence, they mutate their positions until feasible. The procedure ends when no improvement occurs after a certain number of iterations. This whole procedure is represented by a flowchart, as shown in Fig 4.

The model simulation was performed with NetLogo Version 6.2 [22]. The proposed approach uses parallelism in MAS, where each instruction is executed by agents simultaneously. In addition, the platform provides a suitable environment for testing and monitoring the model’s performance.

We compare the results obtained by the PDS-EA model with three heuristics and an exact model. The heuristics used are the bottleneck heuristic (SBH) proposed by Adams et al. [23], sequencing by shortest processing time (SPT), and sequencing by longest processing time (LPT). The exact model is an integer programming problem (IP) [2]. The problem instances were classified according to their scale as small (SS), medium (MS), and large (LS). This classification results from the number of operations, products, and machines. One hundred-two instances from the literature are used (Table 2). The first column of Table 2 shows the authors proposing the test instances. The following columns contain the name of the problem instances and the number of jobs and machines. The last column presents the ranking by the scale of each group of instances. SS contains instances with fewer than 100 operations, MS with more than 100 and fewer than 400 operations, and LS with more than 400 operations. The results of the PDS-EA model with SBH, SPT, LPT, and IP obtained for all test instances are compared, measuring the makespan at different execution times.

**Table 2:** Average (Av.) makespan of the first 10 minutes and best result within 1 hour of execution time for SS, MS, and LS instances.

Inst.	1 min	2 min	3 min	4 min	5 min	6 min	7 min	8 min	9 min	10 min	60 min	Av.^a^
SS	1.21	1.10	1.10	1.06	1.05	1.04	1.04	1.04	1.04	1.04	1.03	1.07
MS	1.34	1.30	1.29	1.27	1.25	1.23	1.22	1.21	1.22	1.20	1.06	1.23
LS	0.85	0.31	0.34	0.40	0.46	0.47	0.50	0.53	0.53	0.53	0.79	0.52

^a^Average value.

The proposed algorithm is compared with three types of standard approaches to the problem. The first approach decomposes the problem into subproblems of less complexity, known as the shifting bottleneck heuristic (SBH) [24]. The second approach considers dispatching rules that are computationally easy to implement because they assign jobs according to the processing time. Specifically, the short processing time (SPT) assigns jobs from shortest to longest, and conversely, the long processing time (LPT) assigns jobs from longest to shortest. In turn, the integer programming (IP)-based method determines the optimal solution of the problem when it is possible according to the available computational resources.

The makespan evaluates the PDS-EA performance by a deviation ratio. Let *m_p_* be the makespan obtained by the PDS-EA and *m_i_* be the makespan obtained by the IP algorithm. The deviation ratio *R* for a runtime of 10 minutes is defined according to Eq 1. In addition, the gap between *m_p_* and the lower bound value known for each instance problem is evaluated.


$$R=\frac{m_{p}}{m_{i}}$$ (1)

## Results

The PDS-EA, on average, matches the results of exact methodologies at medium and low scales and performs better with large-scale instances. Such performance is presented in Table 2, whose first column presents the scales under study, followed by the average, minimum and maximum results for the different execution times. For SS instances, PDS-EA obtains values close to those obtained by the exact method (R≈1). The average for MS instances is 23.8% higher, obtaining the best result with one hour of execution (6% higher than the value of the exact methodology). For LS instances, PDS-ES outperforms IP on average with R = 0.523. In addition, with LS instances, all run times obtain R<1, and the best result occurs at 2 min of execution.

**Fig 5:**
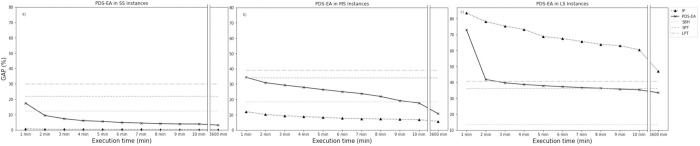
PDS-EA gaps for SS, MS, and LS instances. a. GAP values each minute of simulation Average GAP for SS instances; b. GAP values for each minute of simulation Average GAP for MS instances; c. GAP values each minute of simulation Average GAP for LS instances.

The performance comparison of PDS-EA with IP, SBH, SPT, and LPT is performed with the gap calculated for each instance’s optimal or lower bound value. With small-size instances, IP outperforms the other algorithms. In turn, PDS-EA outperforms all heuristics after 2 minutes of execution. Fig 5A shows the gap for the five methods during one hour of computational time for SS instances. During such a period, PDS-EA evolves gradually, decreasing the makespan difference related to the best-known makespan. After an hour, the exact method is still in process to determine the static optimal solution. Straight lines in the figure represent the heuristic-determined value found immediately at the beginning of the period. The PDS-EA obtains the best result when one hour of computational time is reached. This result differs from the best result found with IP by 3% but improves by 9.08% the best heuristic value obtained with SBH.

With MS instances, the PDS-EA model performs better than the heuristics from minute 10 onward. The best results occur with IP, which outperforms PDS-EA by 4.97% on average. Fig 5B shows the gap for the five methods during one hour of computational time for MS instances. The PDS-EA outperforms the heuristics by 7.58% on average. With the LS instances, the SBH maintains its performance and achieves a gap of less than 20%. This behavior is observed in Fig 5C. Although PDS-EA performs better than the exact method after one hour of computational time, the gaps are larger than for smaller instances. At the end of the period, PDS-EA and IP continue with a decreasing trend, suggesting that convergence is slower for larger instances.

PDS-EA obtains good solutions for the three problem sizes studied. Its main advantage is that the method dynamically optimizes the makespan as time progresses. Fig 5A–5C show that the gradual algorithm produces a better makespan than the heuristic algorithm and the dispatch rules.

**Fig 6:**
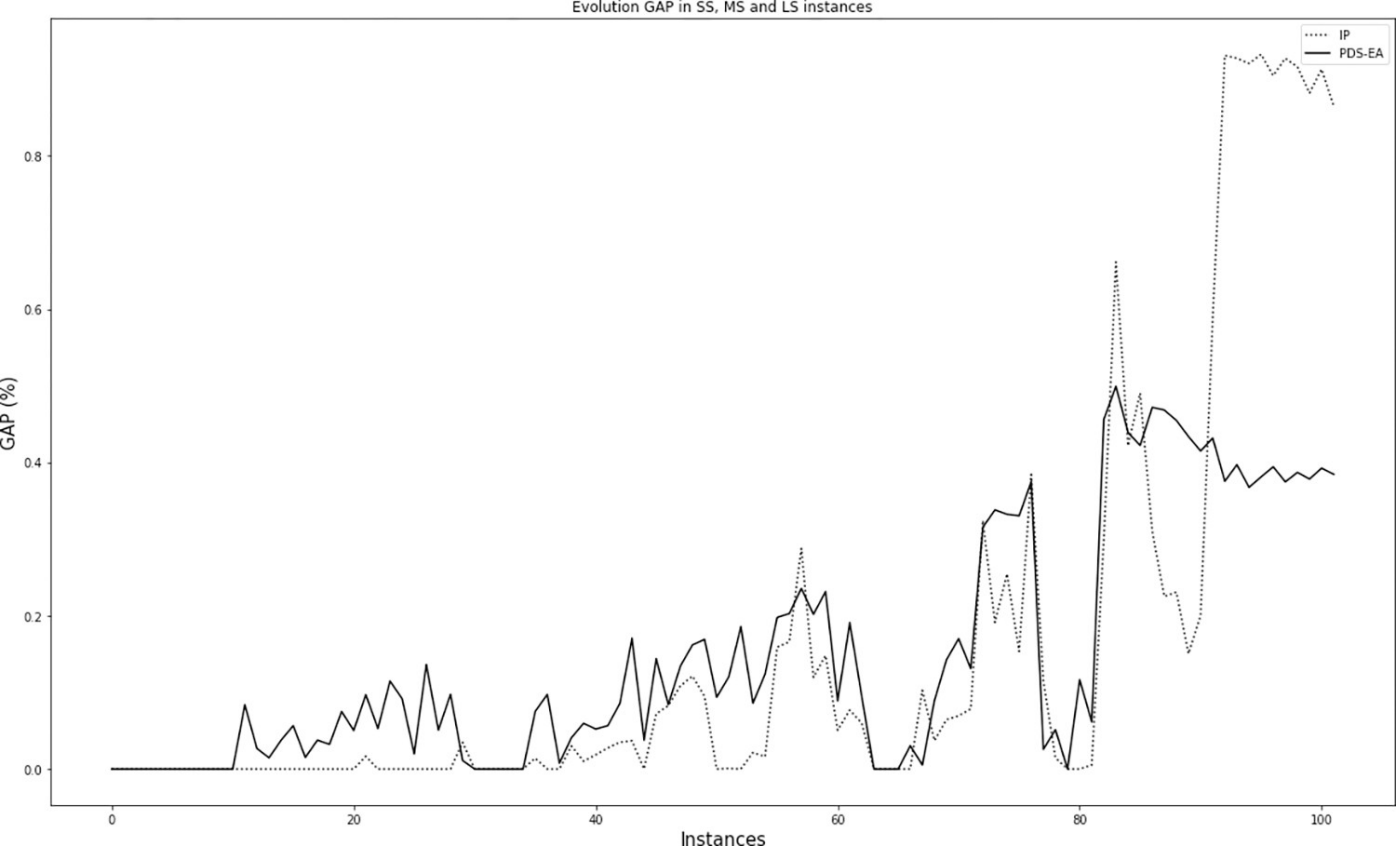
Gap for PDS-EA and IP for all instances ordered by the number of operations.

Although the inclusion of the evolutionary algorithm in the PDS-EA involves an increase in computational time, its performance is maintained for large instances. Fig 5C shows a gradual decrease in the gap to values close to 30% after 1 hour of computational time. Compared to the other sizes, the algorithm requires more time to enter a phase of lower gaps. Even so, it is observed that the process continues to move toward lower gap values, suggesting that a longer simulation time could further reduce the gap. Fig 6 shows the gap obtained in all instances for PDS-EA and IP. A similar behavior is observed when the number of operations is approximately 100. However, for higher numbers, both algorithms have a clear difference. For problems of approximately 300 operations, the difference seems to stabilize.

Computational experiments suggest that the proposed PDS-EA offers competitive results compared to exact and heuristic methods. The PDS-EA architecture generates a stable system capable of reacting to changes without human intervention in short periods. Therefore, PDS-EA can solve sequencing processes in job shop environments at different scales, adapting to the complications of each instance. Thus, PDS-EA positions distributed decision-making as a competitive alternative for job scheduling problems in production systems.

## Conclusion

This paper presents a decentralized decision-making model to minimize the makespan of a job shop problem. The model contemplates a genetic algorithm selecting the best decision at each instant resorting to an agent-based system to simulate the operations. From the flow of products in the different machines, real-time information feeds the model to correct the course of the operations, keeping in mind the minimization of the makespan. The evaluation occurs with a simulation of one hour of computational time, providing the gap concerning the best-known solution for each approach’s small, medium and large-size instances. Data from the literature allow comparison with four standard approaches: an integer programming algorithm and three approximate methods.

The proposed model’s comparative result varies with the number of instances it faces. With small instances, the proposed model underperforms during the whole simulation hour against the exact method, which can find the best solution quickly. With medium-sized instances, a balance is observed between the proposed method and the exact method approaching the hour of simulation time. With large instance sizes, the proposed method outperforms the exact method during that period; however, the SBH rule produces a better solution than all the solutions generated by the proposed method during the hour of simulation time. In turn, despite being very fast in finding the solution, the approximate methods do not present good performance, with small and medium-sized instances being surpassed by the proposed and exact methods.

The proposed model produces near-optimal solutions in a reduced computational time and constitutes an alternative to be computationally implemented to control a manufacturing system. The model uses the holonic-multiagent paradigm for a highly distributed job shop production system. In addition, the model considers dynamic decisions to maintain the system at a production level close to operational optimality.

The proposed model considers the makespan as a performance measure. Several variants of the job shop problem consider other metrics, such as machine utilization rate or task completion delay. The model can be easily modified for such variants, and future studies can explore this field. Also, the model considers decentralized decision-making, and the computational monitoring of the system occurs centrally. It seems natural to consider a parallel computational architecture to perform this task. However, computational parallelism does not always mean better performance. This topic can be explored in future work by identifying the appropriate level of parallelism to improve the model results.

## Supporting information

S1 File (ZIP)

S2 File (ZIP)

S3 File (ZIP)

S4 File (ZIP)

S5 File (ZIP)

S6 File (RAR)

S7 File (ZIP)
